# Mobilisation After Total Knee Replacement Within an Enhanced Recovery Pathway: A Two-Cycle Clinical Audit

**DOI:** 10.7759/cureus.101133

**Published:** 2026-01-08

**Authors:** Maria Pantelidou, Adeolu Adeyeye, Devender Khurana, Elias Pantelidis

**Affiliations:** 1 Ophthalmology, Colchester Hospital, Colchester, GBR; 2 Orthopaedic Surgery, James Paget University Hospital, Great Yarmouth, GBR; 3 Ophthalmology, Ramsay Health Care, Oxford, GBR

**Keywords:** clinical audit, early mobilisation, enhanced recovery after surgery, perioperative care, quality improvement, total knee replacement

## Abstract

Background: Early mobilisation following total knee replacement (TKR) is a key component of Enhanced Recovery After Surgery (ERAS) pathways and is associated with improved functional recovery and reduced length of stay. An initial local audit (MAKE1) identified delays in mobilisation related to postoperative pain and lack of physiotherapy input on postoperative day (POD) 0.

Aim: To assess compliance with early mobilisation standards following implementation of audit recommendations and to identify factors associated with delayed mobilisation and discharge after TKR.

Methods: A retrospective two-cycle clinical audit was conducted in the Orthopaedic Department at James Paget University Hospital. Patients undergoing primary TKR between June and July 2025 were included. Outcomes included time to first mobilisation, length of stay, documented barriers to mobilisation, timing of physiotherapy assessment, and recorded anaesthetic technique. Findings were compared with the initial audit cycle. The audit was registered with the hospital audit department.

Results: A total of 64 patients underwent TKR, and 63 were included in the final analysis due to incomplete documentation for one patient. Mobilisation within 24 hours was achieved in 57/63 patients (90.5%), compared with 35/53 (66%) in the first audit cycle. The median length of stay remained two days. Postoperative pain was the most frequently documented barrier to early mobilisation and was associated with prolonged admission (mean = 5.2 days). Other barriers included delirium, deep vein thrombosis or cellulitis, vasovagal episodes, and fatigue (mean = 4.8 days). No POD 0 physiotherapy assessments were documented; early mobilisation was supported by ward-based staff, with routine physiotherapy review occurring from POD 1. No clear differences in mobilisation timing or discharge were observed between spinal and general anaesthesia.

Conclusion: The descriptive data show higher compliance with mobilisation within 24 hours in MAKE2 compared with MAKE1 following implementation of audit recommendations. Persistent barriers related to pain management and lack of POD 0 physiotherapy input remain key targets for ongoing quality improvement within ERAS pathways.

## Introduction

Enhanced Recovery After Surgery (ERAS) programmes have become integral to perioperative care in elective orthopaedics, including total knee replacement (TKR), improving functional recovery and shortening hospital stay [1,2]. Early mobilisation is a core ERAS principle and has been associated with reduced postoperative complications, improved patient-reported outcomes, and earlier discharge following hip and knee arthroplasty [2-4].

Despite the adoption of standardised ERAS pathways, delays in early mobilisation remain common and may be related to postoperative pain, medical complications, physiotherapy availability, and variability in documentation practices [3,5]. Effective multimodal analgesia and coordinated multidisciplinary input are therefore essential to achieving early mobilisation targets in the immediate postoperative period [1,6].

This two-cycle clinical audit assessed mobilisation within 24 hours following primary TKR and evaluated changes in practice following implementation of audit recommendations. Data were collected across two audit cycles: MAKE1 (April-May 2025) and MAKE2 (June-July 2025). All patients undergoing primary TKR during each audit cycle were included in the study. Recommendations arising from MAKE1 were implemented before MAKE2, which evaluated subsequent mobilisation performance and identified ongoing barriers to recovery.

The MAKE1 audit identified suboptimal compliance with mobilisation within 24 hours after TKR, with key barriers including postoperative pain, symptoms of nausea and hypotension, and limited physiotherapy input on postoperative day (POD) 0. Following implementation of targeted audit recommendations, including reinforcement of existing enhanced recovery practices, improved documentation of reasons for delayed mobilisation, and increased clinical focus on routine management of postoperative pain and symptoms, MAKE2 was conducted to evaluate changes in practice and identify persisting system-level challenges in the early postoperative phase of care.

Objectives

The primary objective of this two-cycle audit was to assess compliance with mobilisation within 24 hours following primary TKR. Secondary objectives were to (1) identify and describe documented barriers to mobilisation within 24 hours, (2) examine whether anaesthetic technique (spinal vs. general anaesthesia) was associated with differences in mobilisation timing, length of stay, or discharge timing within the limits of available documentation, and (3) assess change in practice following implementation of recommendations from the initial audit cycle.

## Materials and methods

Audit design and setting

This study was conducted as a retrospective two-cycle clinical audit within the Orthopaedic Department at James Paget University Hospital, Gorleston, United Kingdom. The audit evaluated compliance with early mobilisation standards following TKR within an enhanced recovery pathway. For the purposes of this audit, mobilisation was defined as the patient being able to stand and take a few steps, with or without a walking aid, typically with assistance from physiotherapy staff and, less commonly, ward-based staff. Mobilisation within 24 hours was assessed using the earliest documented record meeting this definition. As this was a clinical audit/quality improvement project, analysis was descriptive, and no formal statistical hypothesis testing was performed. The project was registered with the hospital audit department and undertaken in accordance with local clinical governance requirements.

Audit recommendations and implementation (MAKE1 → MAKE2)

Following the completion of MAKE1, findings were presented locally and shared with the multidisciplinary team. Recommendations focused on reinforcing existing enhanced recovery practices, improving documentation of reasons for delayed mobilisation, and increasing clinical focus on prompt recognition and routine management/escalation of postoperative symptoms (e.g., pain, nausea, and hypotension) that may delay mobilisation. No new formal pharmacological protocol was introduced as part of this audit; MAKE2 assessed practice after implementation of these audit recommendations within routine clinical care.

Inclusion and exclusion criteria

The audit included all adult patients undergoing primary TKR during the defined audit periods, with no predefined exclusion criteria based on patient characteristics, comorbidities, or perioperative factors. Where individual data items were incomplete or unavailable within the clinical or physiotherapy records, this was documented, and analysis was performed using the available data for the relevant variables. Missing data most commonly related to secondary variables such as anaesthetic technique or documentation of specific reasons for delayed mobilisation, while core outcome measures, including mobilisation timing and length of stay, were available in the clinical records for all patients included in the audit.

Audit standard

The predefined audit standard was mobilisation within 24 hours postoperatively, in keeping with Enhanced Recovery After Surgery (ERAS) principles and local pathway expectations.

Study population

All adult patients undergoing primary TKR during the defined audit periods were included in the audit. Mobilisation timing and length of stay were available for all included patients; where secondary variables were missing (e.g., anaesthetic technique or documented reasons for delayed mobilisation), these were recorded and analysed using available data.

Anaesthetic technique

Anaesthetic technique was categorised as spinal or general anaesthesia based on available documentation. No additional regional nerve blocks were administered to patients undergoing TKR during the audit period. No anaesthesia-specific postoperative analgesic or anti-emetic protocols were evaluated as part of this audit; perioperative symptom management followed standard institutional enhanced recovery practices. Anaesthetic data were collected to explore whether anaesthetic technique was associated with differences in mobilisation timing or discharge outcomes.

Data collection

Data were extracted retrospectively from electronic medical records and physiotherapy documentation. Collected variables included patient demographics (age, sex), time to first mobilisation, length of inpatient stay, timing of physiotherapy assessment (postoperative day zero versus postoperative day one or later), documented reasons for delayed mobilisation where applicable, and recorded anaesthetic technique (spinal or general anaesthesia). Mobilisation timing was recorded as the earliest documented time at which the patient met the audit definition of mobilisation. Physiotherapy documentation was used where available; otherwise, ward/nursing documentation was used. Where times differed between sources, the earliest recorded time was used.

Data organisation and analysis

Audit data were entered into a purpose-designed Microsoft Excel spreadsheet (Microsoft Corporation, Redmond, WA), with each patient recorded as a single entry. Data were organised descriptively, and frequencies, percentages, and summary measures were calculated to allow comparison between the two audit cycles.

Ethics and governance

This project was conducted as a registered clinical audit/service evaluation and did not involve any deviation from standard clinical care. In accordance with the UK Health Research Authority guidance, clinical audits do not require review by a Research Ethics Committee.

## Results

Patient characteristics

In MAKE1, 53 patients underwent primary TKR. In MAKE2, 64 patients underwent primary TKR; 63 were included in the final analysis because documentation was incomplete for one patient. In MAKE1, the mean age was 70.2 years (31 males, 22 females). In MAKE2, the mean age was 70.86 years (25 males, 39 females).

Mobilisation within 24 hours

Mobilisation within 24 hours was achieved in 57/63 patients (90.5%) in MAKE2, compared with 35/53 patients (66.0%) in MAKE1. The median length of stay remained two days in both audit cycles. Key outcomes from the two audit cycles are summarised in Table 1.

**Table 1:** Key outcomes across audit cycles (MAKE1 vs. MAKE2). MAKE2 analysis includes 63 patients due to incomplete documentation for one of 64 TKR procedures. Mobilisation is defined as standing and taking a few steps with or without a walking aid. Abbreviations: TKR, total knee replacement; POD, postoperative day.

Outcome	MAKE1	MAKE2
Audit period	Prior cycle	June–July 2025
Patients analysed (n)	53	63
Mobilised within 24 hours, n (%)	35 (66,0%)	57 (90.5%)
Median length of stay (days)	2	2
Most common barrier to mobilisation	Pain	Pain
POD 0 physiotherapy assessment documented	Limited/inconsistent	0
Anaesthetic technique recorded	Yes	Yes

Barriers to mobilisation

Postoperative pain was the most frequently documented barrier to early mobilisation and was associated with longer admission (mean = 3.4 and 5.2 days) in the two audit cycles. Median time to discharge remained two days in both cycles, and patients who mobilised earlier tended to be discharged sooner. Reasons for delayed mobilisation in MAKE1 included postoperative hypotension and nausea, which were associated with prolonged hospital stay. In MAKE2, other documented factors included one episode of delirium, one episode of deep vein thrombosis or cellulitis, one vasovagal episode, and fatigue. Postoperative nausea and vomiting were less frequently documented in MAKE2. In one case, no reason for delayed mobilisation was recorded.

Physiotherapy assessment

No physiotherapy assessments were documented on POD 0. Physiotherapy input was provided from POD 1 onwards, and patients were typically assessed and mobilised with assistance from physiotherapy staff and, less commonly, ward-based staff. Where mobilisation occurred before physiotherapy review, this was supported by ward-based staff in accordance with the enhanced recovery pathway.

Anaesthetic technique

Of the 63 patients included in the audit, 14 (22%) received general anaesthesia and 48 (76%) received spinal anaesthesia. In one patient (2%), the anaesthetic technique was not documented in the available clinical records. No clear differences in mobilisation timing, length of stay, or discharge outcomes were observed between patients who received general or spinal anaesthesia.

## Discussion

This second audit cycle showed a marked improvement in compliance with mobilisation within 24 hours following TKR, increasing from 66% in the initial audit cycle to over 90% in MAKE2. This improvement suggests that audit-driven feedback and targeted recommendations implemented after MAKE1 were associated with meaningful changes in clinical practice. Early mobilisation is a core component of ERAS pathways and has been consistently linked to improved functional recovery, reduced postoperative complications, and enhanced patient experience following hip and knee arthroplasty [1-4].

The observed improvement is likely multifactorial. Increased staff awareness of mobilisation targets, reinforcement of ERAS principles within the multidisciplinary team, and greater focus on documenting barriers to mobilisation may all have contributed. Audit feedback has been shown to influence clinical behaviour by increasing accountability and promoting reflection on routine practice, particularly when combined with clear, achievable standards. The findings of MAKE2 support the value of structured, iterative audit cycles as an effective quality improvement tool within orthopaedic perioperative pathways.

Despite improved mobilisation performance, the median length of stay remained unchanged at two days. This finding is consistent with existing literature demonstrating that discharge following TKR is influenced by a range of factors beyond early mobilisation alone, including discharge planning processes, patient expectations, social support, and organisational constraints [5,7]. While early mobilisation is a prerequisite for discharge readiness, it may not independently determine length of stay when downstream processes remain unchanged. This highlights the importance of addressing the full discharge pathway, rather than focusing on individual recovery milestones in isolation.

Postoperative pain remained the most frequently documented barrier to early mobilisation and was associated with prolonged admission. Effective pain control is a critical enabler of mobilisation within ERAS pathways, and inadequate analgesia can substantially limit patient engagement with early physiotherapy and mobilisation activities [1,6]. These findings reinforce the importance of optimised multimodal analgesia, proactive pain assessment, and timely escalation to specialist pain services when mobilisation targets are not achieved. Addressing pain-related delays represents a key opportunity to further improve early functional recovery.

A persistent system-level issue identified in MAKE2 was the absence of a documented POD 0 physiotherapy assessment. Early physiotherapy involvement has been shown to enhance patient confidence, promote safe mobilisation, and reinforce ERAS principles during the immediate postoperative period [3,8]. The absence of POD 0 assessment in this audit likely reflects workforce or scheduling constraints rather than deliberate clinical decision-making. This finding highlights a mismatch between ERAS expectations and real-world service capacity, and suggests that pathway optimisation may require organisational change in addition to clinical education. Improving physiotherapy availability and standardising documentation may help ensure that delays to mobilisation are appropriately justified and addressed promptly.

No clear differences were observed in mobilisation timing or discharge outcomes between patients receiving spinal versus general anaesthesia. This aligns with published evidence suggesting that, when ERAS components are effectively implemented, overall recovery trajectories may be influenced more by multidisciplinary perioperative care than by anaesthetic technique alone [2,6]. However, interpretation should remain cautious given the descriptive nature of this audit and incomplete documentation of anaesthetic details in a subset of cases. Physiotherapy service availability (e.g., staffing levels, referral timing, or reasons for non‑attendance on POD 0) was not formally measured; the audit recorded whether the POD 0 physiotherapy assessment was documented. Future work could include service‑level measures to inform workforce planning and pathway redesign.

Taken together, these findings illustrate both the strengths and the ongoing challenges of implementing ERAS principles in routine clinical practice. Overall, this audit highlights the role of continuous quality improvement in enhancing adherence to ERAS principles following TKR. The improvement observed between MAKE1 and MAKE2 is consistent with audit feedback and reinforcement of enhanced recovery practices being associated with improved mobilisation compliance; further cycles will help assess sustainability.

POD 0 physiotherapy was not part of the routine service model during the audit period; introducing POD 0 physiotherapy input is considered likely to be beneficial and represents a potential target for future pathway optimisation. At the same time, the persistence of specific barriers underscores the need for ongoing multidisciplinary engagement, service-level planning, and repeated audit cycles to embed and sustain improvement.

Limitations

This audit is limited by its retrospective design and reliance on the accuracy and completeness of clinical documentation, including incomplete recording of anaesthetic technique and variability in documentation of reasons for delayed mobilisation. Postoperative pharmacological management was not analysed, as this audit focused on audit-driven enhancement of mobilisation practices within routine clinical care rather than evaluation or comparison of specific drug regimens.

As a single-centre audit, the findings reflect local practice; however, the barriers identified and quality improvement strategies described are likely to be relevant to other institutions implementing ERAS pathways for TKR [5,7]. The audit design does not allow causal inference, but is appropriate for assessing compliance with predefined standards and guiding service improvement.

The audit covered a limited time window and sample size, reflecting consecutive cases within routine practice. While sufficient to assess compliance with a predefined standard and guide local improvement, a further cycle (MAKE3) over a longer period would help assess the stability and sustainability of observed changes. Finally, patient-level factors such as the American Society of Anesthesiologists (ASA) grade, BMI, comorbidity burden, preoperative mobility, and postoperative opioid requirements were not systematically captured in this audit dataset and may influence mobilisation and length of stay; future cycles could incorporate these variables to support more granular interpretation.

## Conclusions

The descriptive data show higher compliance with mobilisation within 24 hours in MAKE2 compared with MAKE1 following implementation of audit recommendations. Persistent barriers related to pain management and the absence of POD 0 physiotherapy input remain key priorities for ongoing quality improvement within the enhanced recovery pathway. A further audit cycle (MAKE3) is recommended to evaluate the effectiveness and sustainability of additional targeted interventions.
